# Supplementary material for: Comparison and validation of multiple machine learning algorithms for predicting MDRO infection in catheter-related bloodstream patients: a multicenter cohort study
Source: Microbiol Spectr. 2026 Feb 11;14(3):e03713-25. doi: 10.1128/spectrum.03713-25 (PMC12955437; doi:10.1128/spectrum.03713-25)
Supplement: Tables S1 to S4 — Test for multicollinearity, comparison of performance of each model on training and validation sets, and optimal parameter combination. [file spectrum.03713-25-s0001.docx]

***supplementary material***

**Comparison and Validation of Multiple Machine Learning Algorithms for Predicting MDRO Infection in catheter-related bloodstream Patients: a multicenter cohort study**

Supplementary Table 1. The results of the variance inflation factor (VIF) for each variable in Figure 2d.

Supplementary Table 2. Multi-index line plots of 8 models in the training set (Accuracy, Recall, F1 score, Brier Score).

Supplementary Table 3. Multi-index line plots of 8 models in the validation set (Accuracy, Recall, F1 score, Brier Score).

Supplementary Table 4. The optimal parameters of the eight models.

Supplementary Table 1. The results of the variance inflation factor (VIF) for each variable in Figure 2d.

| **Variable** | **VIF** | **Collinearity Level** |
| --- | --- | --- |
| PT | 76.75 | Severe |
| INR | 76.41 | Severe |
| neu | 3.15 | No |
| Lym2 | 3.03 | No |
| APS3 | 2.33 | No |
| SOFA | 2.22 | No |
| Hb | 1.39 | No |
| albumin | 1.36 | No |
| Pco2 | 1.34 | No |
| PH | 1.28 | No |
| RDW | 1.26 | No |
| BUN | 1.19 | No |
| Ca | 1.18 | No |
| Agap | 1.18 | No |
| SAPS2 | 1.15 | No |
| age | 1.14 | No |
| PC | 1.13 | No |
| K | 1.13 | No |
| Bil | 1.12 | No |
| CRP | 1.12 | No |
| Na | 1.11 | No |
| bas | 1.08 | No |
| Ma | 1.08 | No |
| Admission time | 1.07 | No |
| GLU | 1.07 | No |
| Po2 | 1.06 | No |
| BMI | 1.02 | No |

Supplementary Table 2. Multi-index line plots of 8 models in the training set (Accuracy, Recall, F1 score, Brier Score)

| Model | AUC | Accuracy | Recall | F1 score | Brier Score |
| --- | --- | --- | --- | --- | --- |
| XGBoost | 0.877 | 0.879 | 0.291 | 0.421 | 0.089 |
| RF | 0.852 | 0.819 | 0.248 | 0.370 | 0.114 |
| NN | 0.825 | 0.863 | 0.201 | 0.308 | 0.099 |
| KNN | 0.819 | 0.849 | 0.202 | 0.304 | 0.107 |
| LR | 0.808 | 0.862 | 0.196 | 0.297 | 0.104 |
| LGBM | 0.781 | 0.797 | 0.232 | 0.340 | 0.127 |
| NB | 0.775 | 0.820 | 0.339 | 0.422 | 0.138 |
| SVM | 0.753 | 0.851 | 0.204 | 0.309 | 0.115 |

Supplementary Table 3. Multi-index line plots of 8 models in the validation set (Accuracy, Recall, F1 score, Brier Score).

| Model | AUC | Accuracy | Recall | F1 score | Brier Score |
| --- | --- | --- | --- | --- | --- |
| XGBoost | 0.851 | 0.876 | 0.297 | 0.449 | 0.103 |
| RF | 0.777 | 0.790 | 0.211 | 0.313 | 0.138 |
| NN | 0.677 | 0.760 | 0.190 | 0.286 | 0.155 |
| KNN | 0.601 | 0.728 | 0.202 | 0.285 | 0.169 |
| LR | 0.640 | 0.743 | 0.220 | 0.296 | 0.165 |
| LGBM | 0.820 | 0.797 | 0.230 | 0.340 | 0.135 |
| NB | 0.553 | 0.698 | 0.249 | 0.292 | 0.177 |
| SVM | 0.833 | 0.810 | 0.247 | 0.371 | 0.130 |

Supplementary Table 4.The optimal parameters of the eight models.

| **Model** | **Name of parameter** | **Parameter values** |
| --- | --- | --- |
| XGBoost | max_depth | 3 |
|  | eta | 0.13 |
|  | nrounds | 103 |
|  | subsample | 0.8 |
|  | colsample_bytree | 0.8 |
|  | AUC | 0.794 |
| LR | Number of features | 4 |
|  | intercept term | -2.122 |
|  | AUC | 0.79 |
| RF | ntree | 200 |
|  | mtry | 2 |
|  | AUC | 0.778 |
| SVM | kernel | 2 |
|  | cost | 1 |
|  | AUC | 0.703 |
| LightGBM | nrounds | 100 |
|  | num_leaves | 31 |
|  | AUC | 0.774 |
| NN | size | 0.01 |
|  | decay | 0.718 |
|  | maxit | 0.01 |
|  | AUC | 0.718 |
| KNN | k | 15 |
|  | AUC | 0.736 |
| NB | fL | 0.777 |
|  | AUC | 0.777 |

Abbreviation: LR: LogisticRegression; XGBoost: eXtreme Gradient Boosting; RF:Random Forest; SVM: Support Vector Machine; LightGBM: Light Gradient Boosting Machine; NN: neural networks; KNN: K-NearestNeighbor; NB: Naive Bayes
